# Efeitos Terapêuticos da Tripla Antiagregação Plaquetária em Pacientes Femininas Idosas com Diabetes e Infarto Agudo do Miocárdio

**DOI:** 10.36660/abc.20190442

**Published:** 2021-01-13

**Authors:** Yang Liu, Yanyan Gao, Hengliang Liu, Qi Chen, Jinrui Ji, Kailong Jia

**Affiliations:** 1 Affiliated People’s Hospital of Zhengzhou The Second School of Clinical Medicine Southern Medical University Zhengzhou China Affiliated People’s Hospital of Zhengzhou,The Second School of Clinical Medicine,Southern Medical University, Zhengzhou - China; 2 Shenqiu County Hospital of Traditional Chinese Medicine Shenqiu China Shenqiu County Hospital of Traditional Chinese Medicine, Shenqiu - China

**Keywords:** Agregação Plaquetaria, Acidente Vascular Cerebral, Mulher, Idoso, Diabetes Mellitus, Infarto do Miocárdio, Intervenção Coronária Percutânea/métodos

## Abstract

**Fundamento:**

A dupla antiagregação plaquetária (DAP) é o tratamento fundamental do infarto agudo do miocárdio (IAM).

**Objetivo:**

O presente estudo visou investigar a eficácia e a segurança da tripla antiagregação plaquetária (TAP) em pacientes femininas idosas com diabetes e infarto agudo do miocárdio com supradesnível do segmento ST (IAMCSST), que foram submetidas à intervenção coronária percutânea ICP.

**Métodos:**

Trata-se se de um estudo randomizado e mono-cego. O grupo controle A (97 pacientes idosos do sexo masculino com diabetes e STEMI, cujos escores CRUSADE foram < 30) recebeu aspirina, ticagrelor e tirofibana. Um total de 162 pacientes femininas idosas com diabetes e IAMCSST foram divididas aleatoriamente em dois grupos de acordo com o escore CRUSADE. O grupo B (69 pacientes com escore CRUSADE > 31) recebeu aspirina e ticagrelor. O grupo C (93 pacientes com escore CRUSADE < 30) recebeu aspirina, ticagrelor e tirofibana. Valores de p < 0,05 foram considerados estatisticamente significativos.

**Resultados:**

Após a PCI, o fluxo sanguíneo grau 3 *Thrombolysis in Myocardial Infarction* (TIMI) e a perfusão miocárdica TIMI grau 3 foram significativamente menos prevalentes no grupo B, em comparação com o grupo A (p < 0,05). Quando comparada aos grupos A e C, a incidência de complicações adversas maiores foi significativamente maior no grupo B (p < 0,05).

**Conclusão:**

A TAP pode efetivamente reduzir a incidência de complicações maiores em pacientes idosas com diabetes e IAMCSST. No entanto, atenção cuidadosa deve ser dada à hemorragia em pacientes que recebem TAP. (Arq Bras Cardiol. 2020; [online].ahead print, PP.0-0)

## Introdução

A dupla antiagregação plaquetária (DAP) é um tratamento fundamental do infarto agudo do miocárdio (IAM). Em comparação com o clopidogrel, o efeito inibitório do ticagrelor nas plaquetas no DAPT é rápido e potente, com inibição dupla e combinação reversível. Além disso, pode dilatar as artérias coronárias e é recomendado pelas diretrizes.^1^ No entanto, estudos têm demonstrado que o ticagrelor e o clopidogrel podem aumentar significativamente o risco de hemorragia, em comparação com o uso isolado do clopidogrel.^2^

A incidência de fluxo sanguíneo lento, não-refluxo e complicações trombóticas em pacientes do sexo feminino com diabetes e IAM é maior do que em pacientes sem diabetes ou em pacientes do sexo masculino com diabetes e IAM que estão recebendo DAP.^3 - 5^ Os inibidores dos receptores da glicoproteína IIb/IIIa, em adição à DAP, podem efetivamente reduzir o aparecimento de complicações, tais como fluxo sanguíneo lento, não-refluxo, trombose aguda e subaguda e eventos cardíacos adversos maiores (ECAM).^6 - 9^ Porém, ainda não se sabe se a combinação de medicamentos da tripla antiagregação plaquetária (TAP), especialmente o ticagrelor, aumentaria o risco de hemorragia. A questão urgente no tratamento de IAM é como equilibrar o risco de eventos isquêmicos e de complicações hemorrágicas. Em relação à DAP (aspirina e ticagrelor), o presente estudo teve como objetivo investigar a eficácia e segurança a curto prazo da TAP (aspirina, ticagrelor e tirofibana), em mulheres idosas com diabetes e IAM.

## Materiais e Métodos

### Participantes e Agrupamento

Este estudo foi realizado em dois centros, no Hospital Popular de Zhengzhou da Universidade Médica do Sul e no Hospital de Medicina Tradicional Chinesa do Condado de Shenqiu, ambos na província de Henan. Foram incluídas pacientes femininas idosas com diabetes e infarto agudo do miocárdio com supradesnível do segmento ST (IAMCSST), internadas na unidade coronariana e que receberam intervenção coronária percutânea (ICP) de janeiro de 2013 a dezembro de 2018. O estudo foi randomizado e mono-cego. O sangue foi coletado imediatamente após a admissão. Foi calculado o escore *Can Rapid Risk Stratification of UnsTabela Angina Patients Suppress Adverse Outcomes With Early Implementation of the ACC/AHA Guidelines* (CRUSADE)^10^ dos pacientes de acordo com hematócrito, taxa de depuração da creatinina, frequência cardíaca, pressão arterial, existência de insuficiência cardíaca, diabetes mellitus e doenças vasculares anteriores. Trata-se de um estudo prospectivo. De acordo com o escore CRUSADE, as pacientes foram divididas aleatoriamente em dois grupos: grupo B (pontuação maior que 30) e grupo C (pontuação menor que 30). A DAP (aspirina e ticagrelor) foi administrada às pacientes do grupo B e a TAP (aspirina, ticagrelor e tirofibana) foi administrada às pacientes do grupo C. Pacientes idosos do sexo masculino com diabetes e IAMCSST, que receberam ICP de emergência durante o mesmo período de hospitalização, foram alocados ao grupo A (grupo controle) e receberam a mesma terapia medicamentosa que os pacientes do grupo C. O diagnóstico de IAMCSST foi de acordo com as diretrizes da Sociedade Europeia de Cardiologia^11^ e o diagnóstico de diabetes mellitus foi de acordo com os critérios da Organização Mundial de Sáude.^12^ Foram aplicados os seguintes critérios de inclusão: (1) pacientes com início de IAMCSST em ≤ 12 hours, (2) pacientes com diagnóstico conhecido de diabetes mellitus, (3) pacientes que concordaram em receber ICP de emergência e DAP ou TAP e (4) pacientes com idade entre 60 e 79 anos. Os critérios de exclusão foram os seguintes: (1) pacientes com início de IAMCSST > 12 horas, (2) pacientes com pressão arterial ≥ 180/110 mmHg, (3) pacientes com dissecção aórtica suspeita, (4) pacientes com ICP recuperada após trombólise para IAMCSST, (5) pacientes com histórico de hemorragia cerebral e acidente vascular cerebral isquêmico em um ano, (6) pacientes com insuficiência hepática e/ou renal grave e (7) pacientes com histórico de doenças hemorrágicas.^9^ O presente estudo está de acordo com as normas de ética médica. Com a aprovação do Comitê de Ética do Hospital Popular de Zhengzhou, todos os tratamentos foram realizados com o consentimento esclarecido dos pacientes ou de seus familiares.^9^

### Intervenção Coronária Percutânea de Emergência

Foi registrado eletrocardiograma de dezoito derivações imediatamente após a admissão e os sinais vitais foram verificados. Imediatamente após a admissão, foi coletado o sangue para análise de enzimas cardíacas, troponina e outros itens bioquímicos e de rotina relacionados. Além disso, foram administrados 100 mg de aspirina (Bayer, Alemanha; 100 mg/comprimido) e 180 mg de ticagrelor (Belinda Tabelats, Astra Zeneca; 90 mg/comprimido) por via oral aos pacientes nos três grupos. Foram realizadas angiografia coronária e ICP de emergência. Quando trombos foram encontrados, foi utilizado um cateter de aspiração de trombo para aspirá-los (10 pacientes, 6 pacientes e 9 pacientes nos grupos A, B e C, respectivamente). Foi injetado cloridrato de tirofibana (10 µg/kg) na artéria coronária nos pacientes dos grupos A e C. Após a ICP, 0,075 µg/kg·min de cloridrato de tirofibana foi bombeado continuamente na veia durante 24 horas ^7^. Foram usados stents farmacológicos em todos os pacientes. Apenas o vaso culpado foi tratado durante a emergência. Quando um vaso não culpado precisava de tratamento, foi realizada ICP seletiva 10 a 14 dias depois. Adicionalmente, 100 mg/d de aspirina e 90 mg/d de ticagrelor foram administrados duas vezes por via oral continuamente em pacientes nos três grupos. Posteriormente, a DAP foi aplicada durante pelo menos 12 meses. Além disso, bloqueadores do receptor, estatinas, agentes hipoglicêmicos e inibidores da enzima de conversão da angiotensina foram administrados continuamente.^9^

### Critérios de Observação

Foram coletados e contados os dados para tempo primeiro contato médico-balão, tempo porta-balão, idade, sexo, frequência cardíaca, pressão sistólica, creatinina sérica, classificação Killip da função cardíaca, enzimas miocárdicas, troponina, hematócrito, histórico de doença vascular ou diabetes mellitus e a existência de parada cardíaca. Os escores GRACE^13^ e CRUSADE integral^10^ foram calculados com base nesses dados. Foi calculado o SYNTAX integral^14^ de acordo com as características patológicas da angiografia coronária. Subsequentemente, foram determinadas as características do vaso culpado e foram registrados os dados do diâmetro e do comprimento dos stents. Por exemplo, se mais de dois stents eram necessários para mais de dois vasos culpados, o comprimento dos stents separados era somado para obter o comprimento. Se a lesão-alvo foi mais comprida e mais de dois stents foram colocados em série, 4 mm eram subtraídos do comprimento total. O diâmetro e o comprimento dos stents para cirurgia secundária seletiva de vasos não culpados não foram incluídos. Foram registrados os dados de tempo de internação hospitalar e eventos adversos, incluindo ICP seletiva durante a hospitalização, angina pectoris pós-infarto, re-infarto durante a hospitalização, trombose aguda e subaguda do stent, arritmia grave (taquicardia ventricular persistente, fibrilação ventricular, fibrilação atrial ou flutter atrial hemodinamicamente instável recém-emergido e bloqueio atrioventricular de alto grau, excluindo arritmia de reperfusão durante a ICP), função cardíaca acima de Killip grau III, choque cardiogênico e mortalidade em 30 dias^7 - 9^ dos pacientes dos três grupos. A classificação de sangramento *Thrombolysis in Myocardial Infarction* (TIMI) foi registrada da maneira seguinte: (1) hemorragia grave: hemorragia intracraniana ou sangramento clinicamente visível (incluindo diagnóstico por imagem), diminuição da hemoglobina ≥ 5 g/dl e diminuição do hematócrito ≥ 15%); (2) hemorragia moderada: sangramento clinicamente visível (incluindo diagnóstico por imagem), diminuição da hemoglobina entre 3 e 5 g/dl, sangramento leve; (3) hemorragia leve: sangramento clinicamente visível (incluindo diagnóstico por imagem), com diminuição da hemoglobina < 3 g/dl.^7 - 9 , 15^ Também foram registrados o grau de fluxo sanguíneo TIMI e o grau de perfusão miocárdica TIMI (TMPG) dos vasos relacionados ao infarto após a ICP.^7 - 9 , 15^

### Métodos Estatísticos

Foi usado o software SPSS 17.0 para análise estatística de todos os dados. Os dados de medição, expressos como média ± desvio padrão, apresentaram distribuição normal de acordo com o teste de Kolmogorov-Smirnov. A comparação entre os dois grupos foi realizada pelo teste t de Student não pareado. Os dados de contagem foram expressos em números de casos (razão de constituintes), e a comparação entre os dois grupos foi realizada pelo teste do qui-quadrado. ANOVA de uma via foi utilizada para comparar os três grupos e p < 0,05 foi considerado estatisticamente significativo.^9^

## Resultados

### Características Clínicas

O grupo A (97 pacientes diabéticos do sexo masculino com IAMCSST; idade média: 65,9 ± 9,2 anos), que foi o grupo controle, apresentou escore CRUSADE de baixo risco (inferior a 30). O grupo B (69 pacientes do sexo feminino; idade média: 65,27 ± 9,8 anos) apresentou escore CRUSADE de risco moderado e acima de moderado (superior a 31). O grupo C (93 pacientes do sexo feminino; idade média: 64,8 ± 7,2 anos) apresentou escores CRUSADE de baixo risco (escore inferior a 30). Não houve diferenças estatisticamente significativas em relação a idade, tempo primeiro contato médico-balão, tempo porta-balão, hipertensão, hiperlipidemia, índice de massa corporal, história prévia de ICP, histórico familiar de doença cardíaca coronária e pontuação GRACE entre os três grupos (p *>* 0,05, Tabela 1 ).

**Tabela 1:** – Comparação das características clínicas gerais entre os três grupos

Grupo	Masculino (aspirina + ticagrelor + tirofibana) (Grupo A, 97 casos)	Feminino (aspirina + ticagrelor) (Grupo B, 69 casos)	Feminino (aspirina + ticagrelor + tirofibana) (Grupo C, 93 casos)	p
Idade (anos)	65,9±9,2	65,27±9,8	64,8±7,2	0,824
Sexo (masculino/feminino)	97/0	0/69	0/93	0,000
Histórico de doença do trato digestivo [n(%)]	5(5,15)	3(4,35)	4(4,3)	0,953
Tabagismo [n(%)]	22(22,68)	2(2,9)^a^	3(3,23)^a^	0,000
Etilismo [n(%)]	37(38,14)	7(10,14)	13(13,98)	0,000
FEVE < 40%[n(%)]	9(9,28)	6(8,7)	8(8,6)	0,985
Uso de varfarina [n(%)]	2(2,06)	1(1,45)	2(2,15)	0,943
IBP combinados [n(%)]	6(6,19)	3(4,35)	7(7,53)	0,257
Tempo PCM-B	124,3±67,2	132,5±71,3	128,5±82,6	0,797
Tempo P-B	65,6±21,4	71,3±26,9	62,6±27,2	0,892
Hipertensão [n(%)]	57(58,77)	37(53,62)	55(59,14)	0,745
Hiperlipidemia [n(%)]	49(50,52)	36(52,17)	48(51,62)	0,976
IMC (kg/m^2^)	32,46±4,65	30,13±5,26	29,99±6,31	0,823
Creatinina sérica (mmol/L)	83,29±9,7	96,,32±10,07^a^	83,46±12,35^b^	0,012
Histórico de ICP [n(%)]	4(4,12)	2(2,90)	6(6,46)	0,543
Angina pré-infarto [n(%)]	19(19,59)	2(2,90)^a^	4(4,30)^a^	0,000
Histórico familiar de doença cardíaca coronária [n(%)]	5(5,15)	3(4,35)	5(5,38)	0,954
**Escore GRACE **				
Risco baixo (< 85)[n(%)]	9(9,28)	8(11,59)	9(9,68)	0,526
Risco médio (85 to 133)[n(%)]	17(17,53)	12(17,39)	15(16,13)	0,963
Risco alto (>133)[n(%)]	71(73,20)	49(71,01)	69(74,19)	0,902
**Escore CRUSADE **				
Risco extremamente baixo (1-20 )[n(%)]	37(38,14)	0(0)^a^	42(45,16)^b^	0,000
Risco baixo (21-30)[n(%)]	60(61,86)	0(0)^a^	51(54,84)^b^	0,000
Risco médio (31-40)[n(%)]	0(0)	32(46,38)^a^	0(0)^b^	0,000
Risco alto (41-50)[n(%)]	0(0)	24(34,78)^a^	0(0)^b^	0,016
Risco extremamente alto (>51)[n(%)]	0(0)	13(18,84)^a^	0(0)^b^	0,000

*FEVE: fração de ejeção do ventrículo esquerdo; IBP: inibidores da bomba de prótons; ICP: intervenção coronária percutânea; IMC: índice de massa corporal; P-B: porta-balão; PCM-B: primeiro contato médico-balão. Nota: ^*a*^: p < 0,05 em comparação com o grupo A; ^*b*^: p < 0,05 em comparação com o grupo B.*

### Características das Lesões da Artéria Coronária

Não foram observadas diferenças significativas ao comparar o número de lesões em três artérias coronárias entre os três grupos. No entanto, em comparação com o grupo A, o diâmetro de stent implantado nos grupos B e C foi significativamente menor (p < 0,05). Além disso, o fluxo sanguíneo TIMI grau 3 e o TMPG grau 3 após a ICP foram significativamente menos prevalentes no grupo B do que nos grupos A e C (p *<* 0,05, Tabela 2 ).

**Tabela 2:** – Comparação das características das lesões da artéria coronária entre os três grupos (número de casos, %)

Características das lesões	Masculino (aspirina + ticagrelor + tirofibana) (Grupo A, 97 casos)	Feminino (aspirina + ticagrelor) (Grupo B, 69 casos)	Feminino (aspirina + ticagrelor + tirofibana) (Grupo C, 93 casos)	p
Lesão de ramo único	9(9,28)	6(8,70)	8(8,60)	0,992
Lesão de ramo duplo	11(11,34)	7(10,14)	9(9,68)	0,946
Lesão de ramo triplo	77(79,38)	56(81,16)	75(80,65)	0,956
Complicado com lesão principal esquerda	6(6,19)	3(4,35)	6(6,45)	0,867
Escore SYNTAX	22,21±6,18	21,75±8,57	22,31±7,27	0,863
ICP vaso-alvo Ramo descendente anterior esquerdo	51(52,58)	36(52,17)	49(52,69)	0,998
Ramo circunflexo direito	14(14,43)	11(15,94)	8(8,60)	0,314
Artéria coronária direita	32(32,99)	22(31,88)	36(38,71)	0,564
Diâmetro dos stents (mm, x±s)	3,01±0,33	2,69±0,27^a^	2,70±0,39^a^	0,046
Comprimento dos stents (mm, x±s)	28,29±3,74	27,36±5,13	28,12±5,07	0,783
**Classificação TIMI pré-operativa**				
Grau 0	95(97,94)	68(98,55)	92(98,92)	0,857
Grau 1 ou 2	2(2,06)	1(1,45)	1(1,08)	0,957
Grau 3	0	0	0	
**Classificação TIMI pós-operativa**				
Graus 0 a 2	2(2,06)^c^	10(14,49)^ac^	4(4,30)^bc^	0,003
Grau 3	95(97,94)^c^	59(85,51)^ac^	89(95,70)^bc^	0,003
**Classificação TMPG pré-operativa**				
Graus 0 a 2	97(100)	69(100)	93(100)	
Grau 3	0	0	0	
**Classificação TMPG pós-operativa**				
Graus 0 a 2	6(6,19)	20(28,99)^ad^	14(15,05 )^abd^	0,0003
Grau 3	91(93,81)^d^	49(71,01)^ad^	79(84,95)^abd^	0,0001

*ICP: intervenção coronária percutânea. Nota: ^*a*^: p < 0,05 em comparação com o grupo A; ^*b*^: p < 0,05 em comparação com o grupo B; ^*c*^: comparações com TIMI pré-operatório intra-grupo do mesmo grau, p < 0,05; ^*d*^: comparações com TMPG pré-operatório intra-grupo do mesmo grau, p < 0,05.*

### Tempo de Internação Hospitalar, Características da ICP e Incidência de Complicações

O tempo médio de internação hospitalar foi significativamente mais alto no grupo B que nos grupos A e C (p *<* 0,05). Além disso, o grupo B teve 14 casos de angina pectoris pós-infarto, enquanto os outros dois grupos tiveram 5 casos cada. Ocorreram 9 casos de arritmia grave no grupo B, o que foi maior do que nos outros dois grupos (2 casos no grupo A e 3 casos no grupo C). Houve 14 casos de insuficiência cardíaca, choque cardiogênico e mortalidade em 30 dias no grupo B, o que foi maior do que nos grupos A e C (5 e 4 casos, respectivamente). Além disso, a incidência de ICP seletiva, re-infarto e trombose de stent durante a internação hospitalar foi significativamente mais alta no grupo B, em comparação com o grupo A (p *<* 0,05). No grupo B, ocorreram 14 casos de angina pectoris pós-infarto, 7 casos de re-infarto e 3 casos de trombose de stent. Em adição a isso, a incidência total de hemorragia e a incidência de hemorragia moderada foram significativamente maiores no grupo C, quando comparados aos grupos A e B (p *<* 0,05, Tabela 3 ). Houve 8 casos de hemorragia moderada no grupo C, enquanto os grupos A e B tiveram 1 caso cada.

**Tabela 3:** – Comparação de tempo de internação hospitalar, características da ICP e incidência de complicações nos três grupos

Item	Masculino (aspirina + ticagrelor + tirofibana) (Grupo A, 97 casos)	Feminino (aspirina + ticagrelor) (Grupo B, 69 casos)	Feminino (aspirina + ticagrelor + tirofibana) (Grupo C,93 casos)	p
Tempo médio de internação hospitalar (dias)	7,8±1,5	11,2±3,3^a^	8,3±1,9^ab^	0,042
Cirurgia secundária seletiva [n(%)]	12(12,37)	19(27,54)^a^	9(20,93)	0,047
Angina pectoris pós-infarto [n(%)]	5(5,05)	14(19,72)^a^	5(5,38)^b^	0,001
Re-infarto [n(%)]	0(0)	7(10,14)^a^	6(6,45)	0,009
Trombose de stent [n(%)]	0(0)	3(4,35)^a^	1(1,08)	0,073
Arritmia grave [n(%)]	2(2,06)	9(13,04)^a^	3(3,23)^b^	0,004
Função cardíaca acima de Killip grau III [n(%)]	2(2,60)	14(20,29)^a^	8(8,60)^ab^	0,0001
Choque cardiogênico pós-operatório [n(%)]	0(0)	5(7,25)^a^	1(1,08)^b^	0,006
Mortalidade em 30 dias [n(%)]	0(0)	4(5,80)^a^	1(1,08)	0,004
Hemorragia total [n(%)]	7(7,22)	6(8,70)	20(21,98)^ab^	0,005
Hemorragia grave	0(0)	0(0)	1(1,08)	0,408
Hemorragia moderada	1(1,03)	1(1,45)	8(8,60)^ab^	0,012
Hemorragia leve	6(6,19)	5(7,25)	11(11,83)	0,344

*a: p < 0,05 em comparação com o grupo A; b: p < 0,05 em comparação com o grupo B.*

## Discussão

Mais de 50% das mortes cardiovasculares ocorrem em mulheres. Independentemente dos resultados positivos da fisiopatologia, queixas, sintomas, sinais e resultados dos exames auxiliares, ou dos efeitos terapêuticos de curto e longo prazo, as pacientes do sexo feminino apresentam as suas particularidades.^16 , 17^ A ICP oportuna em pacientes do sexo feminino com síndrome coronariana aguda pode efetivamente reduzir a incidência de ECAM.^9 , 15 , 17 , 18^ No entanto, as mulheres, especialmente as idosas, geralmente não apresentam dor torácica típica durante um ataque isquêmico, o que pode atrasar o diagnóstico e o tratamento. No presente estudo, a incidência de angina pectoris antes do infarto foi significativamente menor nas mulheres em comparação com os homens. Além disso, sintomas não específicos, bem como desconforto pós-esternal, aperto no peito, falta de ar, náusea, vômito e fadiga, foram mais comuns em mulheres com IAM. Em adição a isso, a sensibilidade e a especificidade do eletrocardiograma e do eletrocardiograma de exercício no diagnóstico de doença cardíaca coronária são menores em mulheres. A falta de sintomas específicos e as alterações eletrocardiográficas correspondentes no quadro fisiopatológico de isquemia e a falta de medicamentos e intervenções no estilo de vida oportunas e necessárias, mesmo em condições críticas, como oclusão vascular completa ou subtotal, frequentemente levam a consulta tardia, perdendo a fase mais tratável e resultando em falta de intervenção precoce, como a ICP de emergência; isto é uma das principais causas da maior incidência de complicações e mortalidade em pacientes do sexo feminino com IAM.^17 , 18^

A diabetes mellitus é uma condição isócrita de doença cardíaca coronária. Os efeitos terapêuticos dos medicamentos antiplaquetários em pacientes com diabetes são piores do que em pacientes sem diabetes. Além disso, a incidência de fluxo sanguíneo lento e não-refluxo após a ICP de emergência e as ECAM são significativamente maiores do que em pacientes sem diabetes.^7 - 9 , 15^ Isto sugere que uma terapia de antiagregação plaquetária intensificada é necessária para pacientes com diabetes. Diabetes mellitus é um dos fatores de risco mais importantes para pacientes com síndrome coronariana aguda, independentemente de o escore GRACE ser usado para avaliar eventos isquêmicos ou o escore CRUSADE ser usado para predizer o risco de sangramento. A DAP é o tratamento fundamental para a prevenção de isquemia e trombose após a ICP.^19^ Após receber a DAP, alguns pacientes ainda apresentam complicações graves de trombose, especialmente pacientes do sexo feminino com diabetes e pacientes idosos com diabetes. Os inibidores do receptor IIb/IIIa, em adição à DAP, podem efetivamente reduzir a ocorrência de fluxo sanguíneo lento e não-refluxo, a incidência de trombose aguda e subaguda e a ocorrência de ECAM.^7 - 9 - 15^ No entanto, não houve diferenças significativas em relação às características gerais, às características da lesão coronariana e aos escores GRACE entre os três grupos do presente estudo. Esses resultados revelaram que a TAP foi significativamente superior à DAP na redução de eventos relacionados à isquemia, como a incidência de angina pectoris pós-infarto, arritmia grave, insuficiência cardíaca, choque cardiogênico e mortalidade em 30 dias, em pacientes idosos de ambos os sexos com diabetes e IAMCSST, quando comparados a mulheres com diabetes e IAMCSST que receberam DAP. Além disso, em comparação com as pacientes que receberam DAP, a permanência hospitalar média foi menor naqueles que receberam a TAP. Em adição a isso, a incidência de ICP seletiva, re-infarto e trombose de stent em pacientes idosos masculinos com diabetes e IAMCSST que receberam TAP foi significativamente menor, quando comparada com as pacientes femininas idosas com diabetes e IAMCSST que receberam a DAP. Isso sugere que a TAP poderia efetivamente reduzir o início de eventos isquêmicos em pacientes idosos com diabetes e IAMCSST. Além disso, os pacientes do sexo masculino se beneficiam mais do que os do sexo feminino.

O escore CRUSADE é um índice importante para avaliar o risco de hemorragia ^10^. A idade avançada, a presença de diabetes mellitus e o sexo feminino são todos fatores de risco importantes para a hemorragia. Devido ao forte efeito antiplaquetário da tirofibana e à aplicação combinada de ticagrelor, as complicações hemorrágicas sempre foram motivo de preocupação por parte dos médicos cardiovasculares.^3 , 7 - 9 , 15^ Atenção especial deve ser dada à aplicação de DAP e TAP para minimizar a ocorrência de complicações hemorrágicas. A DAP tem sido aplicada apenas para pacientes femininas idosas com IAMCSST, cujo escore CRUSADE estava acima do risco médio, enquanto a TAP foi aplicada para pacientes com escores CRUSADE de baixo risco. Esses resultados revelaram que a incidência total de hemorragia e a incidência de hemorragia moderada em pacientes idosas com diabetes após a TAP foi significativamente maior, quando comparada àquela de pacientes do sexo feminino com risco relativamente alto de hemorragia e pacientes idosos do sexo masculino com diabetes recebendo TAP. Isto indicou que o risco de hemorragia é maior para pacientes femininas idosas com diabetes e IAMCSST. Vale ressaltar que existem condições fisiopatológicas especiais na terapia antiplaquetária para pacientes idosas com diabetes e IAMCSST, que são diferentes das do sexo masculino. Ao prevenir eventos causados por isquemia, atenção especial deve ser dada ao aumento do risco de hemorragia.

### Limitações do Estudo

O estudo comparou apenas pacientes femininas idosas com diabetes que receberam DAP e TAP e não incluiu pacientes do sexo masculino recebendo DAP como controle. Além disso, o tamanho da amostra era pequeno e houve falta de acompanhamento a longo prazo. A equipe de autores está conduzindo pesquisas adicionais para resolver essas limitações.

## Conclusão

O presente estudo mostrou que a incidência de complicações em mulheres idosas com diabetes e IAMCSST que receberam TAP após ICP foi significativamente menor do que em pacientes que receberam DAP. No entanto, a incidência de hemorragia nas pacientes femininas que receberam TAP foi significativamente maior, quando comparada aos pacientes masculinos que receberam TAP e às pacientes femininas que receberam DAP.
