# Supplementary material for: Efeitos Terapêuticos da Tripla Antiagregação Plaquetária em Pacientes Femininas Idosas com Diabetes e Infarto Agudo do Miocárdio
Source: Arq Bras Cardiol. 2021 Jan 13;116(2):229–35. [Article in Portuguese] doi: 10.36660/abc.20190442 (PMC7909981; doi:10.36660/abc.20190442)
Supplement: Translation

# Translation: Therapeutic Effects of Triple Antiplatelet Therapy in Elderly Female Patients with Diabetes and Acute Myocardial Infarction

**Authors:** Yang Liu, Yanyan Gao, Hengliang Liu, Qi Chen, Jinrui Ji, Kailong Jia

**Affiliations:** 1 China Affiliated People’s Hospital of Zhengzhou,The Second School of Clinical Medicine,Southern Medical University, Zhengzhou - China; 2 China Shenqiu County Hospital of Traditional Chinese Medicine, Shenqiu - China

**Keywords:** Platelet Aggregation, Stroke, Woman, Aging, Diabetes Mellitus, Myocardial Infarction, Percutaneous Coronary Intervention/methods

## Abstract

**Background:**

Dual antiplatelet therapy (DAPT) is the cornerstone treatment of acute myocardial infarction (AMI).

**Objective:**

The present study aimed to investigate the efficacy and safety of triple antiplatelet therapy (TAPT) in elderly female patients with diabetes and ST segment elevation myocardial infarction (STEMI), who had undergone percutaneous coronary intervention (PCI).

**Methods:**

We designed a randomized, single-blind study. Control group A (97 elderly male patients with diabetes and STEMI, whose CRUSADE scores were < 30) received aspirin, ticagrelor, and tirofiban. A total of 162 elderly female patients with diabetes and STEMI were randomly divided into two groups according to CRUSADE score. Group B (69 patients with CRUSADE score > 31) received aspirin and ticagrelor. Group C (93 patients with CRUSADE score < 30) received aspirin, ticagrelor and tirofiban. P values < 0.05 were considered statistically significant.

**Results:**

Compared to the findings in group A, post-PCI Thrombolysis in Myocardial Infarction (TIMI) grade 3 blood flow and TIMI myocardial perfusion grade 3 were significantly less prevalent in group B (p < 0.05). When compared to groups A and C, the incidence of major adverse complications was significantly higher in group B (p < 0.05).

**Conclusion:**

TAPT could effectively reduce the incidence of major complications in elderly female patients with diabetes and STEMI. However, close attention should be paid to hemorrhage in patients receiving TAPT. (Arq Bras Cardiol. 2020; [online].ahead print, PP.0-0)

## Introduction

Dual antiplatelet therapy (DAPT) is a cornerstone in the treatment of acute myocardial infarction (AMI). Compared with clopidogrel, the inhibitory effect of ticagrelor on platelets in DAPT is rapid and potent, with dual-inhibition and reversible combination. Furthermore, it can dilate coronary arteries, and it is recommended by guidelines.^1^However, studies have shown that ticagrelor and clopidogrel might significantly increase the risk of hemorrhage, compared with clopidogrel.^2^

The incidence of slow blood flow, no-reflow, and thrombotic complications in female patients with diabetes and AMI is higher than in patients without diabetes or in male patients with diabetes and AMI who are receiving DAPT.^3 - 5^Glycoprotein IIb/IIIa receptor inhibitors, in addition to DAPT, can effectively reduce the onset of complications, such as slow blood flow, no reflow, acute and subacute thrombosis, and major adverse cardiac events (MACE).^6 - 9^However, it remains unknown whether the combination of triple antiplatelet therapy (TAPT) drugs, especially ticagrelor, would increase the risk of hemorrhage. The urgent issue in the treatment of AMI is how to balance the risk of ischemic events and hemorrhagic complications. In relation to DAPT (aspirin and ticagrelor), the present study aimed to investigate the short-term efficacy and safety of TAPT (aspirin, ticagrelor, and tirofiban) in elderly female patients with diabetes and AMI.

## Materials and Methods

### Subjects and Grouping

This study was conducted in two centers, Zhengzhou People’s Hospital of Southern Medical University and Shenqiu County Hospital of Traditional Chinese Medicine, both in the Henan province. Elderly female patients with diabetes and STEMI, who were admitted to the coronary care unit and who received emergency percutaneous coronary intervention (PCI) treatment from January 2013 to December 2018, were included into this study. This study was randomized and single-blinded. Blood was drawn immediately after admission. The Can Rapid Risk Stratification of Unstable Angina Patients Suppress Adverse Outcomes With Early Implementation of the ACC/AHA Guidelines (CRUSADE) score^10^was calculated according to patients’ hematocrit, creatinine clearance rate, heart rate, blood pressure, existence of heart failure, diabetes mellitus, and previous vascular diseases. This was a prospective study. According to CRUSADE score, patients were randomly divided into the following two groups: group B (score higher than 30) and group C (score lower than 30). DAPT (aspirin and ticagrelor) was given to the patients in group B, and TAPT (aspirin, ticagrelor, and tirofiban) was given to the patients in group C. Elderly male patients with diabetes and STEMI, who received emergency PCI during the same period of hospitalization, were assigned to group A (control group), and they received the same drug therapy as the patients in group C. Diagnosis of STEMI was in accordance with the European Society of Cardiology guidelines,^11^and diagnosis of diabetes mellitus was in accordance with the World Health Organization criteria.^12^The inclusion criteria were as follows: (1) patients with the onset of STEMI at ≤ 12 hours, (2) patients with known diagnosis of diabetes mellitus, (3) patients who agreed to receive emergency PCI and DAPT or TAPT, and (4) patients between the ages of 60 and 79 years old. The exclusion criteria were as follows: (1) patients with the onset of STEMI at > 12 hours, (2) patients with blood pressure ≥ 180/110 mmHg, (3) patients with suspected aortic dissection, (4) patients with retrieved PCI after thrombolysis for STEMI, (5) patients with a history of cerebral hemorrhage and ischemic stroke within one year, (6) patients with severe hepatic and/or renal insufficiency, and (7) patients with a history of hemorrhagic diseases.^9^The present study conforms to the standards of medical ethics. With the approval of the Ethics Committee of Zhengzhou People’s Hospital, all treatments were performed with the informed consent of patients or their families.^9^

### Emergency Percutaneous Coronary Intervention

Eighteen-lead electrocardiogram was recorded immediately after admission, and vital signs were checked. Blood was drawn immediately after admission for assay of cardiac enzymes, troponin, and other related biochemical and routine testing items. In addition, 100 mg of aspirin (Bayer, Germany; 100 mg/tablet) and 180 mg of ticagrelor (Belinda Tablets, Astra Zeneca; 90 mg/tablet) were orally administered to patients in these three groups. Emergency coronary angiography and PCI were performed. If thrombi were found, a thrombus aspiration catheter was used to aspirate them (10 patients, 6 patients and 9 patients in groups A, B, and C, respectively). Tirofiban hydrochloride (10 µg/kg) was injected into the coronary artery in patients in groups A and C. After PCI, 0.075 µg/kg·min of tirofiban hydrochloride was continuously pumped into the vein for 24 hours^7^. Drug-eluting stents were used for all patients. Merely the culprit vessel was treated during the emergency. If a non-culprit vessel needed to be treated, selected PCI was performed 10 to 14 days later. In addition, 100 mg/d of aspirin and 90 mg/d of ticagrelor were given twice orally and continuously in patients in all three groups. Subsequently, DAPT was applied for at least 12 months. Furthermore, -receptor blockers, statins, hypoglycemic agents, and angiotensin converting enzyme inhibitors were continuously administered.^9^

### Observation Criteria

The data for first-medical-contact-to-balloon time, door-to-balloon time, age, sex, heart rate, systolic pressure, serum creatinine, Killip classification of cardiac function, myocardial enzymes, troponin, hematocrit, past history of vascular disease and diabetes mellitus, and the existence of cardiac arrest were collected and counted. The GRACE scores^13^and CRUSADE integral^10^were calculated based on the above data. The SYNTAX integral^14^was calculated according to the pathological characteristics of coronary angiography. Subsequently, the characteristics of the culprit vessel were determined. Data for diameter and length of stents were recorded. For instance, if more than two stents were needed for more than two culprit vessels, the length of the separate stents was added to obtain the length of stent. If the target lesion was longer and more than two stents were placed in series, the total length of the stent was subtracted by 4 mm. The diameter and length of stents for selective secondary surgery of non-culprit vessels were not included. Data were also recorded for length of stay and adverse events, including selective PCI during hospitalization, post-infarction angina pectoris, re-infarction during hospitalization, acute and sub-acute thrombosis in the stent, severe arrhythmia (persistent ventricular tachycardia, ventricular fibrillation, newly emerged hemodynamically unstable atrial fibrillation or atrial flutter, and high grade atrioventricular block, excluding reperfusion arrhythmia during PCI), cardiac function above Killip grade III, cardiogenic shock, and 30-day mortality^7 - 9^of patients in these three groups. The Thrombolysis in Myocardial Infarction (TIMI) bleeding classification was recorded as follows: (1) severe hemorrhage: intracranial hemorrhage or clinically visible bleeding (including imaging diagnosis), decreased hemoglobin of ≥ 5 g/dl and decreased hematocrit by ≥ 15%); (2) moderate hemorrhage: clinically visible bleeding (including imaging diagnosis), decreased hemoglobin between 3 and 5 g/dl, slight bleeding; (3): mild hemorrhage: clinically visible bleeding (including imaging diagnosis), with decreased hemoglobin < 3 g/dl.^7 - 9 , 15^TIMI blood flow grading and TIMI myocardial perfusion grade (TMPG) of the infarction-related vessels after PCI were also recorded.^7 - 9 , 15^

### Statistical Methods

SPSS 17.0 software was used for statistical analysis of all data. The measurement data were expressed as mean ± standard deviation. They presented a normal distribution according to Kolmogorov-Smirnov test, and the comparison between the two groups was conducted by unpaired Student’s t test. The count data was expressed as the number of cases (constituent ratio), and the comparison between the two groups was performed by chi-squared test. One-way ANOVA was employed to compare these three groups, and p < 0.05 was considered statistically significant.^9^

## Results

### Clinical Characteristics

Group A (97 male patients with diabetes and STEMI; mean age: 65.9 ± 9.2 years), which was the control group, had low risk CRUSADE scores (less than 30). Group B (69 female patients; mean age: 65.27 ± 9.8 years) had moderate and above moderate risk CRUSADE scores (higher than 31). Group C (93 female patients; mean age: 64.8 ± 7.2 years) had low risk CRUSADE scores (lower than 30). There were no statistically significant differences in age, first-medical-contact-to-balloon time, door-to-balloon time, hypertension, hyperlipidemia, body mass index, previous history of PCI, family history of coronary heart disease, and GRACE score among the three groups (p *>* 0.05, Table 1 ).

**Table 1:** – Comparison of the general clinical characteristics among the three groups

Group	Male (aspirin + ticagrelor + tirofiban) (Group A, 97 cases)	Female (aspirin + ticagrelor) (Group B, 69 cases)	Female (aspirin + ticagrelor + tirofiban) (Group C, 93 cases)	p
Age (years)	65.9±9.2	65.27±9.8	64.8±7.2	0.824
Sex (male/female)	97/0	0/69	0/93	0.000
History of digestive tract disease [n(%)]	5(5.15)	3(4.35)	4(4.3)	0.953
Smoking [n(%)]	22(22.68)	2(2.9)^a^	3(3.23)^a^	0.000
Alcohol consumption [n(%)]	37(38.14)	7(10.14)	13(13.98)	0.000
LVEF < 40%[n(%)]	9(9.28)	6(8.7)	8(8.6)	0.985
Warfarin use [n(%)]	2(2.06)	1(1.45)	2(2.15)	0.943
Combined PPI [n(%)]	6(6.19)	3(4.35)	7(7.53)	0.257
FMC-to-B time	124.3±67.2	132.5±71.3	128.5±82.6	0.797
D-to-B time	65.6±21.4	71.3±26.9	62.6±27.2	0.892
Hypertension [n(%)]	57(58.77)	37(53,62)	55(59.14)	0.745
Hyperlipidemia [n(%)]	49(50.52)	36(52.17)	48(51.62)	0.976
BMI (kg/m^2^)	32.46±4.65	30.13±5.26	29.99±6.31	0.823
Serum creatinine (mmol/L)	83.29±9.7	96..32±10.07^a^	83.46±12.35^b^	0.012
History of PCI [n(%)]	4(4.12)	2(2.90)	6(6.46)	0.543
Pre-infarction angina [n(%)]	19(19.59)	2(2.90)^a^	4(4.30)a	0.000
Family history of coronary heart disease [n(%)]	5(5.15)	3(4.35)	5(5.38)	0.954
**GRACE score**				
Low risk (< 85)[n(%)]	9(9.28)	8(11.59)	9(9.68)	0.526
Middle risk (85 to 133)[n(%)]	17(17.53)	12(17.39)	15(16.13)	0.963
High risk (>133)[n(%)]	71(73.20)	49(71.01)	69(74.19)	0.902
**CRUSADE score**				
Extremely low risk (1-20 )[n(%)]	37(38.14)	0(0)a	42(45.16)^b^	0.000
Low risk (21-30)[n(%)]	60(61.86)	0(0)a	51(54.84)^b^	0.000
Middle risk (31-40)[n(%)]	0(0)	32(46.38)^a^	0(0)^b^	0.000
High risk (41-50)[n(%)]	0(0)	24(34.78)^a^	0(0)^b^	0.016
Extremely high risk (>51)[n(%)]	0(0)	13(18.84)^a^	0(0)^b^	0.000

*BMI: body mass index; D-to-B: door-to-balloon; FMC-to-B: first-medical-contact-to-balloon; LVEF: left ventricular ejection fraction; PCI: percutaneous coronary intervention; PPI: proton pump inhibitors. Note:^*a*^: p < 0.05 compared with group A;^*b*^: p < 0.05 compared with group B.*

### Characteristics of Coronary Artery Lesions

No significant differences were observed when comparing the number of lesions in three coronary arteries among the three groups. However, compared to group A, the diameter of the implanted stent in groups B and C was significantly smaller (p *<* 0.05). In addition, TIMI grade 3 blood flow and TMPG grade 3 after PCI were significantly less prevalent in group B than in groups A and C (p *<* 0.05, Table 2 ).

**Table 2:** – Comparison of the characteristics of coronary artery lesions among the three groups (number of cases, %)

Lesion characteristics	Male (aspirin + ticagrelor + tirofiban) (Group A, 97 cases)	Female (aspirin + ticagrelor) (Group B, 69 cases)	Female (aspirin + ticagrelor + tirofiban) (Group C, 93 cases)	p
Single branch lesion	9(9.28)	6(8.70)	8(8.60)	0.992
Double branch lesion	11(11.34)	7(10.14)	9(9.68)	0.946
Three branch lesion	77(79.38)	56(81.16)	75(80.65)	0.956
Complicated with left main lesion	6(6.19)	3(4.35)	6(6.45)	0.867
SYNTAX score	22.21±6.18	21.75±8.57	22.31±7.27	0.863
PCI Target vessel Left anterior descending branch	51(52.58)	36(52.17)	49(52.69)	0.998
Right circumflex branch	14(14.43)	11(15.94)	8(8.60)	0.314
Right coronary artery	32(32.99)	22(31.88)	36(38.71)	0.564
Diameter of stents (mm, x±s)	3.01±0.33	2.69±0.27^a^	2.70±0.39^a^	0.046
Length of stents (mm, x±s)	28.29±3.74	27.36±5.13	28.12±5.07	0.783
**Preoperative TIMI classification**				
Grade 0	95(97.94)	68(98.55)	92(98.92)	0.857
Grade 1 or 2	2(2.06)	1(1.45)	1(1.08)	0.957
Grade 3	0	0	0	
**Postoperative TIMI grading**				
Grade 0 to 2	2(2.06)^c^	10(14.49)^ac^	4(4.30)^bc^	0.003
Grade 3	95(97.94)^c^	59(85.51)^ac^	89(95.70)^bc^	0.003
Preoperative TMPG classification				
Grade 0 to 2	97(100)	69(100)	93(100)	
Grade 3	0	0	0	
**Postoperative TMPG grading**				
Grade 0 to 2	6(6.19)	20(28.99)^ad^	14(15.05 )^abd^	0.0003
Grade 3	91(93.81)^d^	49(71.01)^ad^	79(84.95)^abd^	0.0001

*PCI: percutaneous coronary intervention. Note:^*a*^: p < 0.05 compared with group A;^*b*^: p < 0.05 compared with group B;^*c*^: comparisons with intra-group preoperative TIMI of the same grade, p < 0.05;^*d*^: comparisons with intra-group preoperative TMPG of the same grade, p < 0.05.*

### Length of Hospital Stay, Characteristics of PCI, and Incidence of Complications

The average length of hospital stay was significantly higher in group B than in group A and C (p *<* 0.05). In addition, group B had 14 cases of post-infarction angina pectoris, whereas the other two groups had 5 cases each Nine cases of severe arrhythmia occurred in group B, which was higher than that in other two groups (2 cases in group A and 3 cases in group C). There were 14 cases of heart failure, cardiogenic shock, and 30-day mortality in group B, which was higher than in groups A and C (5 cases and 4 cases, respectively). Furthermore, the incidence of selective PCI, re-infarction, and stent thrombosis during hospitalization was significantly higher in group B, when compared to group A (p *<* 0.05). In group B, there were 14 cases of post-infarction angina pectoris, 7 cases of re-infarction and 3 cases of stent thrombosis. Moreover, the total incidence of hemorrhage and the incidence of moderate hemorrhage were significantly higher in group C, when compared to groups A and B (p *<* 0.05, Table 3 ). There were 8 cases of moderate hemorrhage in group C, whereas groups A and group B had 1 case each.

**Table 3:** – Comparison of length of hospital stay, characteristics of PCI, and incidence of complications in the three groups

Item	Male (aspirin + ticagrelor + tirofiban) (Group A, 97 cases)	Female (aspirin + ticagrelor) (Group B, 69 cases)	Female (aspirin + ticagrelor + tirofiban) (Group C,93 cases)	p
Average length of hospital stay (days)	7.8±1.5	11.2±3.3^a^	8.3±1.9^ab^	0.042
Selective secondary surgery [n(%)]	12(12.37)	19(27.54)^a^	9(20.93)	0.047
Post-infarction angina pectoris [n(%)]	5(5.05)	14(19.72)^a^	5(5.38)^b^	0.001
Re-infarction [n(%)]	0(0)	7(10.14)^a^	6(6.45)	0.009
Stent thrombosis [n(%)]	0(0)	3(4.35)^a^	1(1.08)	0.073
Severe arrhythmia [n(%)]	2(2.06)	9(13.04)^a^	3(3.23)^b^	0.004
Cardiac function above Killip grade III [n(%)]	2(2.60)	14(20.29)^a^	8(8.60)^ab^	0.0001
Postoperative cardiogenic shock [n(%)]	0(0)	5(7.25)^a^	1(1.08)^b^	0.006
30-day mortality [n(%)]	0(0)	4(5.80)^a^	1(1.08)	0.004
Total hemorrhage [n(%)]	7(7.22)	6(8.70)	20(21.98)^ab^	0.005
Severe hemorrhage	0(0)	0(0)	1(1.08)	0.408
Moderate hemorrhage	1(1.03)	1(1.45)	8(8.60)^ab^	0.012
Mild hemorrhage	6(6.19)	5(7.25)	11(11.83)	0.344

*a: p < 0.05 compared with group A; b: p < 0.05 compared with group B.*

## Discussion

More than 50% of cardiovascular deaths occur in females. Regardless of the positive results of the pathophysiology, complaints, symptoms, signs and results of auxiliary examinations, or the short-term and long-term therapeutic effects, female patients have their own particularities.^16 , 17^Timely PCI in female patients with acute coronary syndrome can effectively reduce the incidence of MACE.^9 , 15 , 17 , 18^However, women, especially elderly women, often have no typical chest pain during ischemic attack, which may delay diagnosis and treatment. In the present study, the incidence of angina pectoris before infarction was significantly lower in women, when compared to men. Furthermore, non-specific symptoms, such as post-sternal discomfort, chest tightness, shortness of breath, nausea, vomiting, and fatigue, were more common in females with AMI. In addition, the sensitivity and specificity of electrocardiogram and exercise electrocardiogram in the diagnosis of coronary heart disease are lower in women. The lack of specific symptoms and the corresponding electrocardiogram changes in the pathophysiological condition of ischemia and the lack of timely and necessary medicines and lifestyle intervention, even in critical conditions, such as complete or subtotal vascular occlusion, often lead to late consultation, missing the most treatable stage and resulting in a lack of early intervention, such as emergency PCI, which is one of the main causes of the higher incidence of complications and mortality in female patients with AMI.^17 , 18^

Diabetes mellitus is an isocritical condition of coronary heart disease. The therapeutic effects of antiplatelet drugs in patients with diabetes are worse than in patients without diabetes. Furthermore, the incidence of slow blood flow and no reflow after emergency PCI and MACE are significantly higher than in patients without diabetes.^7 - 9 , 15^This suggests that enhanced antiplatelet therapy is needed for patients with diabetes. Diabetes mellitus is one of the most important risk factors for patients with acute coronary syndrome, regardless of whether the GRACE score is used for evaluating ischemic events or the CRUSADE score is used for predicting the risk of bleeding. DAPT is the cornerstone for preventing ischemia and thrombosis after PCI.^19^After receiving DAPT, some patients still have serious complications of thrombosis, especially in female patients with diabetes and elderly patients with diabetes. IIb/IIIa receptor inhibitors in addition to DAPT can effectively reduce the occurrence of slow blood flow and no reflow, the incidence of acute and subacute thrombosis, and the occurrence of MACE.^7 - 9 - 15^However, there were no significant differences in general characteristics, coronary lesion characteristics and GRACE scores among the three groups in the present study. These results revealed that TAPT was significantly superior to DAPT in reducing related ischemia-driven events, such as the incidence of post-infarction angina pectoris, severe arrhythmia, heart failure, cardiogenic shock, and 30-day mortality, in both elderly male and female patients with diabetes and STEMI, when compared to women with diabetes and STEMI who received DAPT. Furthermore, compared to patients receiving DAPT, the average hospital stay was shorter in those receiving TAPT. Moreover, the incidence of selective PCI, re-infarction, and stent thrombosis in elderly male patients with diabetes and STEMI who received TAPT was significantly lower, when compared to elderly female patients with diabetes and STEMI who received DAPT. This suggested that TAPT could effectively reduce the onset of ischemic events in elderly patients with diabetes and STEMI. Furthermore, male patients benefit more than female patients.

The CRUSADE score is an important index for evaluating the risk of hemorrhage.^10^Older age, the existence of diabetes mellitus, and female sex are all important risk factors for hemorrhage. Due to the strong antiplatelet effect of tirofiban and the combined application of ticagrelor, hemorrhagic complications have always been a matter of concern for cardiovascular physicians.^3 , 7 - 9 , 15^Close attention should be given to the application of DAPT and TAPT in order to minimize the occurrence of hemorrhagic complications. DAPT has been applied only for elderly female patients with STEMI, whose CRUSADE score was above middle-risk, while TAPT has been applied for patients with low-risk CRUSADE scores. These results revealed that the total incidence of hemorrhage and the incidence of moderate hemorrhage in elderly female patients with diabetes after TAPT was significantly higher, when compared to that of female patients with relatively high risk of hemorrhage and elderly male patients with diabetes receiving TAPT. However, there was no significant difference in the risk of hemorrhage between male and female patients receiving TAPT. This indicated that the risk of hemorrhage is higher for elderly female patients with diabetes and STEMI. It is noteworthy that there are special pathophysiological conditions in antiplatelet therapy for elderly female patients with diabetes and STEMI, which are different from those of males. While preventing ischemia-driven events, close attention must be given to the increased risk of hemorrhage.

### Study Limitations

The study only compared elderly female patients with diabetes who received TAPT and DAPT, and it did not include male patients receiving DAPT as a control. Furthermore, the sample size was small, and there was a lack of long-term follow-up. The team of authors is conducting further research to address these limitations.

## Conclusion

The present study showed that the incidence of complications in elderly female patients with diabetes and STEMI receiving TAPT after PCI was significantly lower than in patients receiving DAPT. However, the incidence of hemorrhage in female patients receiving TAPT was significantly higher, when compared to male patients receiving TAPT and female patients receiving DAPT.
